# Identification of Putative Plant-Based ALR-2 Inhibitors to Treat Diabetic Peripheral Neuropathy

**DOI:** 10.3390/cimb44070194

**Published:** 2022-06-29

**Authors:** Mohd Saeed, Munazzah Tasleem, Ambreen Shoib, Mohd Adnan Kausar, Abdel Moneim E. Sulieman, Nadiyah M. Alabdallah, Zeina El Asmar, Abdelmuhsin Abdelgadir, Asma Al-Shammary, Md Jahoor Alam, Riadh Badroui, Maryam Zahin

**Affiliations:** 1 Department of Biology, College of Sciences, University of Ha’il, P.O. Box 2240, Ha’il 81451, Saudi Arabia; lhadi@hotmail.com (A.M.E.S.); zeinaelasmar@gmail.com (Z.E.A.); abdelmuhsin@yahoo.com (A.A.); j.alam@uoh.edu.sa (M.J.A.); badraouir@yahoo.fr (R.B.); 2 School of Electronic Science and Engineering, University of Electronic Science and Technology of China, Chengdu 610054, China; munazzah.t@gmail.com; 3 Department of Clinical Pharmacy, College of Pharmacy, Jazan University, P.O. Box 114, Jazan 45142, Saudi Arabia; as@gmail.com; 4 Department of Biochemistry, College of Medicine, University of Ha’il, P.O. Box 2240, Ha’il 81451, Saudi Arabia; mak@gmail.com; 5 Department of Biology, College of Science, Imam Abdulrahman Bin Faisal University, P.O. Box 1982, Dammam 31441, Saudi Arabia; nmalabdallah@iau.edu.sa; 6 Department of Public Health, College of Public Health and Health Informatics, University of Ha’il, P.O. Box 2240, Ha’il 81451, Saudi Arabia; dr.asma.alshammary@gmail.com; 7 Section of Histology-Cytology, Medicine Faculty of Tunis, University of Tunis El Manar, Tunis 1007, Tunisia; 8 James Graham Brown Cancer Center, University of Louisville, Louisville, KY 4020, USA; maryam.zahin@louisville.edu

**Keywords:** pharmacophore, structure-based drug design, NuBBE_DB_, ADMET, molecular docking

## Abstract

Diabetic peripheral neuropathy (DPN) is a common diabetes complication (DM). Aldose reductase -2 (ALR-2) is an oxidoreductase enzyme that is most extensively studied therapeutic target for diabetes-related complications that can be inhibited by epalrestat, which has severe adverse effects; hence the discovery of potent natural inhibitors is desired. In response, a pharmacophore model based on the properties of eplarestat was generated. The specified pharmacophore model searched the NuBBE_DB_ database of natural compounds for prospective lead candidates. To assess the drug-likeness and ADMET profile of the compounds, a series of in silico filtering procedures were applied. The compounds were then put through molecular docking and interaction analysis. In comparison to the reference drug, four compounds showed increased binding affinity and demonstrated critical residue interactions with greater stability and specificity. As a result, we have identified four potent inhibitors: ZINC000002895847, ZINC000002566593, ZINC000012447255, and ZINC000065074786, that could be used as pharmacological niches to develop novel ALR-2 inhibitors.

## 1. Introduction

Diabetes Mellitus (DM) is a health disorder that is rapidly becoming an epidemic in several countries. A sedentary lifestyle, unhealthy food, obesity, and being overweight are major factors. Saudi Arabia’s economy has changed dramatically in the previous four decades. Prosperity and expansion have changed people’s lifestyles [1,2,3]. Notably, eating habits and physical activities have deteriorated. Cars, elevators, escalators, and remotes have reduced activity. Traditional reliance on locally farmed fruits, vegetables, and wheat has changed. All these factors have contributed to a dramatic increase in diabetes prevalence in Saudi Arabia [1]. DM is a common endocrine disease with numerous micro- and macrovascular consequences. Diabetic peripheral neuropathy (DPN) has long been a research focus as it is one of the most common consequences of diabetes mellitus (DM). Diabetic complications are caused by the upregulation of the polyol and hexosamine pathways and increase the amounts of non-enzymatic glycation products and protein kinase C activity [2]. Pathological characteristics of DPN, a common consequence of chronic DM, include axonal atrophy, demyelination, and the delayed regeneration of peripheral sensory nerve fibers. The pathophysiology of DPN in dysfunctional peripheral nerve repair and regeneration is not yet clearly known [3]. These issues have a complicated etiology and can manifest in a variety of ways. ALR-2 is a rate-limiting enzyme in the polyol pathway that belongs to the oxidoreductase family and is the most intensively researched therapeutic target for treating diabetes-related consequences [4].

The overactivation of the ALR-2 enzyme in the polyol pathway has also been linked to an imbalance in the NADPH/NADP^+^ and NADH/NAD^+^ ratios, which increases oxidative stress by lowering reduced glutathione levels (GSH) [5,6]. Oxidative stress is characterized by increased reactive oxygen species formation and impaired antioxidant defenses due to an imbalance between oxidative components and antioxidant capabilities [7]. Diabetic problems are exacerbated by oxidative stress [8]. To guide the treatment, it is vital to understand how antioxidant defenses differ in diabetic peripheral neuropathy. The polyol pathway, which is a part of metabolizing glucose, contains an essential enzyme known as aldose reductase [9]. Diabetic hyperglycemia promotes the polyol pathway, which uses aldose reductase to convert glucose to sorbitol. Although fructose reductase can convert sorbitol to fructose, the lack of fructose kinase in peripheral nerve tissue induces intracellular hypertonia and restricts inositol absorption [2]. As a direct result of this, aldose reductase inhibitors have been studied for use as potential therapies for patients suffering from DPN. Epalrestat is an aldose reductase inhibitor that is marketed in Japan, China, and India. Unlike conventional diabetic neuropathy medications, epalrestat may abate disease progression. Animal studies have shown that epalrestat reduces sorbitol in the sciatic nerve, erythrocytes, ocular tissues, and human erythrocytes. Taking 50 mg of epalrestat three times a day improved motor and sensory nerve conduction velocity and subjective neuropathy symptoms compared to baseline and placebo [10]. The Food and Drug Administration (FDA) and the European Medicines Agency (EMA) have not approved it for use in the United States, and the reason for this is that the only large multicenter study that assessed its efficacy and safety was an open-label study. [11]. Several adverse effects of epalrestat include: cerebral infarction, dorsal pruritus, eczema, rash, skin eruption, diarrhoea, discomfort, nausea, vomiting, increased liver enzyme levels, increased serum creatinine level, edema, hand stiffness, hot flashes, lightheadedness, thirst, numbness, vertigo, and lower-extremity weakness [10,12,13].

Natural products have multidimensional chemical structures, which has sparked interest in their use as biological function modifiers. They have influenced chemicobiology and have been used to discover new drugs. Physical chemistry has revealed natural products’ structural diversity. Complex three-dimensional chemical and steric properties of the natural compound enable the efficiency and selectivity of molecular targets. Drug development has benefited from natural product research. Various drugs derived from natural products are being used in treatment successfully. For instance, artemisinin and its analogs are used as anti-malarial compounds; *Vinca alkaloids* from *Catharanthus roseus* and the *terpene paclitaxel* from *Taxus baccata* are among successful anti-cancer drugs and various anti-hypertensive drugs [4,5]. Due to their chemical diversity and novel mechanisms of action, natural products have been used in many drug development and research programs. Natural products have evolved to interact with a wide range of biological targets, and some have become important drugs in healthcare [6,7]. The natural product database was used in this work to look for new candidates that might be used as potent and selective ALR-2 inhibitors with a higher therapeutic index and lesser side effects. Pharmacophoric properties of a reference drug must be found to include in new hits while screening a database. Since epalrestat is useful in diabetic neuropathy in clinical investigations, this research aimed to find small molecules that demonstrated epalrestat’s pharmacophoric properties. Pharmacophore modeling is the best method for carrying out this strategy. Thus, single ligand-based pharmacophore modeling was used for the hierarchical virtual screening, scoring of ligands based on the LUDI function, drug-likeness and ADMET characteristics estimates, docking, and intra-molecular interaction studies to identify the most potent natural compounds to treat DPN. Furthermore, by employing in vitro and in vivo models using epalrestat as a reference, these hits can be empirically confirmed for their therapeutic characteristics.

## 2. Material and Methodology

### 2.1. Structure-Based Pharmacophore Model Generation

The structure-based pharmacophore model (SBPM) has been successfully employed in many therapeutic domains to create new drugs with significant biological activity [14]. ALR-2 X-ray crystal structure with epalrestat was adopted from RCSB Protein Data Bank [15]; PDB ID: 4JIR, resolved at 2.0 Å with a molecular weight of 37.15 kDa and 316 amino acid residues in a single unique chain [16]. During the study, the implicated pharmacophore characteristic was predicted using the bound inhibitor-Epalrestat. SBPM was generated in this study utilizing the Biovia Discovery Studio (DS) module Interaction Pharmacophore Generation [Dassault Systems, BIOVIA Corp., San Diego, CA, USA, v 21.1] based on the co-crystal structure of ALR-2 complexed with the epalrestat. The analysis used a binding site sphere with a radius of 12 Å, which covered all the key residues in the binding pocket of ALR-2, and the bound inhibitor-eplarestat. This SBPM procedure uses the bioactive conformation of a drug to generate a selective pharmacophore model from a single ligand. This technique was created to evaluate the protein–ligand interaction at the binding pocket, which results in the pharmacophoric properties of hydrogen bond acceptor (HBA), hydrogen bond donor (HBD), hydrophobic (H), negative ionizable (NI), positive ionizable (PI), and aromatic ring (RA). Additionally, to improve the effectiveness of virtual screening, the excluded volumes were taken into account when creating pharmacophore models [17]. This technique lists all the pharmacophoric properties, scores all possible pharmacophore combinations, and finally determines the best pharmacophore. A total of 10 hypothesis models were created, with the best one being designated as Hypo 1. The highly selective pharmacophore model was used to screen the NuBBE_DB_ [8].

### 2.2. Screening Hits and Enzyme Crystal Structure

Phytochemicals from Nuclei of Bioassays, Ecophysiology, and Biosynthesis of Natural Products Database (NuBBE_DB_) were used in this study. NuBBE_DB_ was established in 2013 as a comprehensive compendium of available biogeochemical knowledge about Brazilian biodiversity and has shown to be a significant resource for new drug design and dereplication research with a larger diversity of natural sources. The NuBBE_DB_ contains validated multidisciplinary information, chemical descriptors, species origins, geographic locations, spectroscopic data (NMR), and pharmacological properties and is freely accessible online (https://nubbe.iq.unesp.br/portal/nubbedb.html, last accessed on 8 November 2021) [18].

### 2.3. Scoring the Screened Compounds

Virtual screening relies heavily on scoring and ranking docked ligands. The optimum scoring function should be employed to increase the chances of success. LUDI scoring function was applied to score the natural compounds. LUDI is a scoring program from DS (Dassault Systems, BIOVIA Corp., San Diego, CA, USA, v 21.1) that places small molecules in the active protein site so that hydrogen bonds can be formed and hydrophobic pockets can be filled. A 3D structure of the protein-inhibitor complex is often utilized to suggest novel substituents for an existing inhibitor. LUDI can link fragments to existing ligands and fit them into interaction sites [19,20,21].

### 2.4. Drug Likeness and ADMET Analysis

Virtually screened natural compounds that passed the LUDI scoring function were selected for in silico drug-likeness and ADME-Toxicity (ADMET) calculations using Filter by Lipinski and Veber Rules Module ADMET Descriptors and TOPKAT module from DS. Calculations were performed on parameters like the log of the n-octanol/water partition coefficient (LogP), the molecular weight (MW), the number of hydrogen bond acceptors (HBA), the number of hydrogen bond donors (HBs), the molecular polar surface area (PSA), and the number of rotatable bonds (RotB) that were included in Lipinski’s rule of five and Veber rule [22,23].

### 2.5. Molecular Docking and Interaction Studies

Molecular docking with DS’s CDOCKER Module (Dassault Systems, BIOVIA Corp., San Diego, CA, USA, v 21.1) was used to further validate the compounds that best fit the resulting pharmacophore model. This was done so that the most promising molecules could be identified. CDOCKER is a method based on molecular dynamics that employs simulated annealing [24]. Before starting the docking protocol, the selected compounds were prepared using the “Prepare Ligands” technique from DS to eliminate duplicates, synthesize 3D conformations, and exclude compounds with undesired features before molecular docking. Followed by protein preparation, the receptor protein was cleaned initially, with undesirable crystal structures, water molecules, and other bounded ligands removed. The receptor protein’s quality was evaluated, then loop refinement was performed, and the receptor protein was validated for the existence of disallowed regions. In CDOCKER, “Ligand Partial Charge Method” was set to “CFF”, and “Input Site Sphere” was set to “−6.01145, 8.69569, and 17.4568” in x, y, and z coordinates, respectively, with a Pose Cluster Radius of 0.1 Å in the binding pocket of ALR-2. For each molecule, just one top docking pose was reported and stored for subsequent study. We determined the RMSD values between the optimal ligand docking poses and the conformations in co-crystal structures. Close intra-molecular interactions between the selected natural compounds and the active site residues were evaluated to assess the binding affinity and stability of the complex [25,26].

## 3. Results and Discussions

Every pharmacological molecule’s pharmacokinetics (PK) and pharmacodynamic (PD) phases demonstrate its biological response. The in silico investigation of epalrestat-based potential ALR-2 inhibitors is included in this study, which takes into account multiple molecular events in both the PK and PD phases. The in silico PD investigation comprises pharmacophore modeling, virtual screening for lead discovery, molecular docking, and intra-molecular interactions of the hits with ALR-2 to determine potency. The in silico PK analysis involves extensive drug-likenesss and ADMET profiling of retrieved hits using a variety of DS’s basic criteria and tools.

### 3.1. Structure-Based Pharmacophore Model Generation

**Figure 1:**
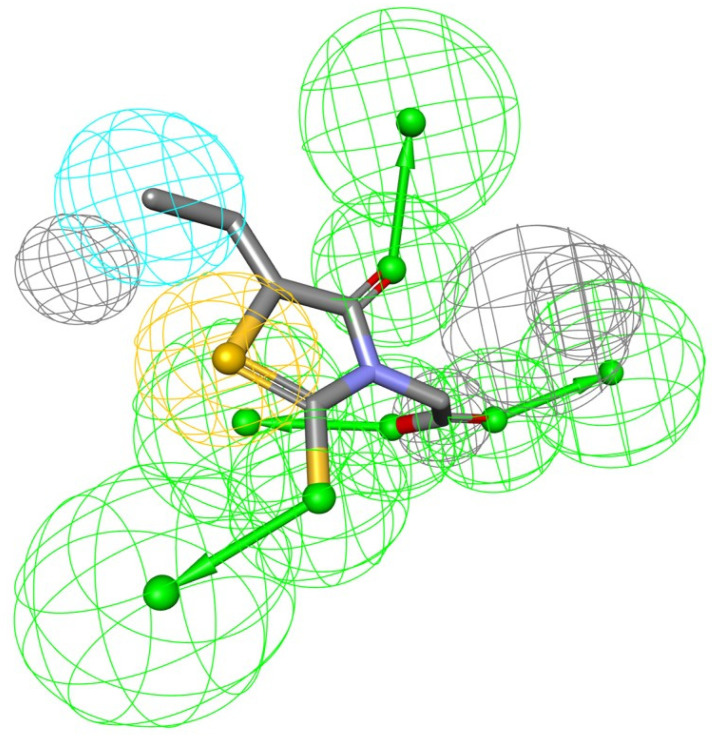
Generated pharmacophore model exploiting eplarestat.

The biological activity of a substance is influenced not only by its physical properties, but also by its three-dimensional conformation. It is a pharmacophore that includes the steric and electronic features necessary for supramolecular interactions with a specific biological target and the triggering (or blocking) of its biological response. According to this approach, bioactive compounds’ interactions with their targets are depicted as a 3D arrangement of abstract properties rather than specific functional groups. Hydrogen bonding, charged interactions, metal interactions, and hydrophobic and aromatic contacts are some examples. Many pharmacophore modeling programs allow steric limitations. The exclusion volumes replicate the geometry of the binding pocket and prohibit the mapping of inactive substances due to protein surface conflicts [9]. As a result, the bioactive conformation of epalrestat was derived from a high-resolution X-ray crystal structure of ALR-2 complexed with epalrestat from RCSB Protein Data Bank (PDB ID: 4JIR). Pharmacophores of the epalrestat were generated using Interaction Pharmacophore Generation from DS. Utilizing the epalrestat, the auto pharmacophore was generated, which resulted in 10 pharmacophore models with the same features, such as HBA, H, and sulfur interaction when the minimum inter feature distance was set as 2, minimum features were set to 4, and maximum features as 6. Subsequently, 10 pharmacophore models were generated. The first model was chosen as it aligned well with the known inhibitor-eplarestat, and demonstrated four HBA, one H, and one sulfur interaction, as illustrated in Figure 1.

The use of pharmacophore modeling in computational drug development identifies new candidate compounds that exhibit the important qualities represented by a pharmacophore model. As a result, the current research adapts to identify the major important characteristics described by the epalrestat and ALR-2 interaction.

### 3.2. Screening and Scoring the Natural Compound Database

To identify the most potent natural compound, 40,000 natural compounds from NuBBE_DB_ were screened. The virtual screening of NuBBE_DB_ using pharmacophore 1 model yielded 34 hits, as shown in Table 1. These hits, when scored based on LUDI scoring function from DS, were found to be in the range of 237 to 1328, indicating the high quality of the selected pharmacophore model. Some of the compounds were found to score higher than the eplarestat, as shown in Table 2.

### 3.3. Drug Likeness and ADMET Analysis

**Figure 2:**
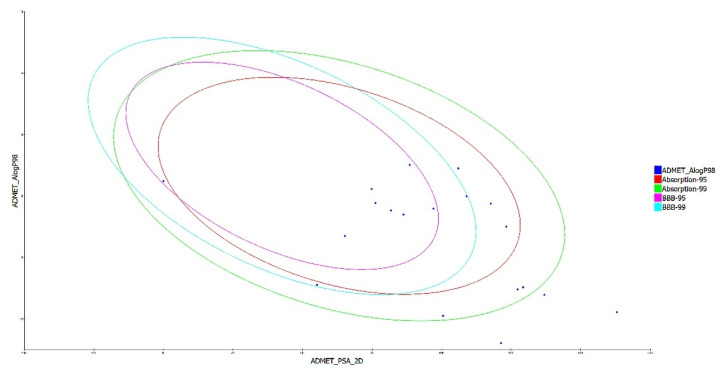
A plot of polar surface area (PSA) versus ALogP in ADMET analysis of filtered substances, with % and % confidence limit ellipses corresponding to the blood–brain barrier (BBB) and intestinal absorption, respectively.

The drug-like characteristics of the virtually screened natural compounds were evaluated using the Lipinski and Veber rule. Compounds qualifying two or more of the Lipinski and Veber rule were evaluated for drug-like qualities to achieve better results. Our findings also demonstrated that 19 natural compounds out of 34 virtually screened compounds qualified for the drug-likeness parameter, having entirely fulfilled Lipinski’s rule of five, which specifies that a molecule cannot breach more than two of the following parameters (MW < 500 Da, LogP < 5, HBD < 5, and HBA < 10) for it to be utilized safely as a therapeutic agent, as shown in Table 3 of the ADMET analysis. The ADMET properties of a molecule play a significant role in drug discovery; these attributes are primarily responsible for medicine failure in around 60% of clinical trial cases [27]. A molecule with a favorable ADME profile is absorbed through the gastrointestinal system and available in circulation, processed by metabolic enzymes and eliminated from the body, and does not interfere with normal biological processes [22]. The ADMET descriptors module in DS evaluates AlogP98, PSA (polar surface area), plasma protein binding (PPB), hepatotoxicity, CYP2D6 enzyme inhibition, aqueous solubility blood-brain barrier (BBB), and intestinal absorption of a drug-like molecule. In water at 25 °C, a linear regression model was used to predict aqueous solubility. After oral administration, the compounds’ absorption and solubility levels indicate human intestine absorption and drug likeliness. For intestinal absorption, the values should be ≥0, where 0 signifies good absorption, or 1 (moderate absorption), and for aqueous solubility, 3 signifies good and 4 is optimal [28]. The hydrophilicity of a compound is determined by its AlogP98 value, with AlogP98 > 5 indicating high absorption or permeability. PSA is another important factor that influences drug bioavailability. Compounds having a PSA ≤ 140 Å are able to be absorbed passively, and as a result, they have a high oral bioavailability [10,11,12]. Most of the filtered compounds showed high absorption and good aqueous solubility. Using 2D PSA and AlogP98 descriptors with 95% and 99% confidence ellipses, the ADME model predicted intestinal absorption and blood–brain barrier penetration. The region enclosed within the ellipses defines the well-absorbed compounds [29]. The current investigation predicted 8 out of 19 substances with good absorption within the 99% confidence ellipse, as shown in Figure 2. The BBB level measures the quantity of drug penetration into the central nervous system (CNS) after oral delivery. A desirable drug would not breach the BBB since it could negatively affect the CNS. As a result, therapeutic molecules with BBB values of 3 or 2 (low or medium) are usually thought to be the best for administration [27].

The hepatotoxicity level of a drug compound can be determined by its potential to cause dose-dependent liver damage, and drug toxicity is usually anticipated using this information. CYP450 enzymes and isoforms regulate drug metabolism. Inhibiting these detoxifying enzymes can produce toxicity [30]. CYP2D6 accounts for 2% of the overall CYP content, yet it biotransforms 20% of hepatically metabolized pharmaceuticals [22]. More than 80% of clinical trial medicines are metabolized by five CYP isoforms (3A4, 2D6, 2C19, 2C9, and 1A2). None of the drugs inhibited the CYP2D6 enzyme in this investigation, and no severe drug interaction toxicity was observed in the liver. The PPB is a measure of how well a medicine binds to blood proteins. The drug’s efficacy may be influenced by the degree to which it binds. The PPB values were categorized into “false” and “true” for “poorly” and “highly bounded” drug molecules, respectively, as shown in Table 4.

TOPKAT is a widely used technique for evaluating drug candidates’ potential ecotoxicity, toxicity, mutagenicity, and reproductive or developmental toxicity [13]. The results of TOPKAT and ADMET demonstrate that the anticipated carcinogenicity values of the filtered compounds are within the expected range, and there is no risk of mutagenicity. A few chemicals, however, induce mild skin irritation, and mild-to-severe eye irritation, and they may cause developmental or reproductive toxicity if administered long-term or at higher dosages. Table 5 summarizes other toxicity screening characteristics such as Rat inhalation LC_50_, Rat of Oral LD_50_, Fathead minnow LC_50_, and Daphnia EC_50_. Most of the filtered compounds were found to be non-carcinogenic, non-mutagenic, non-toxic, with mild-to-moderate skin and ocular irritancy, and degradable. ZINC000012447255 showed a higher Rat Oral LD_50_ and Rat Inhalation LC_50_ score than eplarestat, indicating its lesser toxicity.

### 3.4. Molecular Docking and Interaction Studies

Molecular docking has emerged as a valuable computational tool for the virtual screening of drug candidates. It paves the door for faster drug development by evaluating the activity of a large number of compounds against target proteins and providing information on candidate ligand–protein interactions in a short period of time, reducing the cost of laboratory-based screening [31]. The top four virtually screened compounds having high LUDI scores and qualifying the drug likeness and ADMET analysis were considered to evaluate the binding affinity with receptor protein ALR-2. The top four natural compounds that were obtained using the virtual screening procedures were then advanced to the molecular docking studies. The purpose of these studies was to analyze the binding affinities and intra-molecular interactions between the protein and the discovered compounds, and therefore to eliminate any false positives [32]. Molecular docking studies also define the predicted binding modes of the ligand at the protein active site. The CDOCKER software, included with the DS, was used to dock the top four natural compounds into the ALR-2 binding site using the CHARMm-based molecular docking approach, resulting in random ligand conformations when employing high-temperature molecular dynamics (MD). CDOCKER is a grid-based docking technique that utilizes the CDOCKER algorithm to fine-tune docking for a particular receptor protein against many ligands. High-temperature molecular dynamics and random rotations yield random ligand conformations during molecular docking. Grid-based (GRID 1) simulated annealing explores random rotations. The random rotations are further investigated using grid-based (GRID 1) simulated annealing.

**Figure 3:**
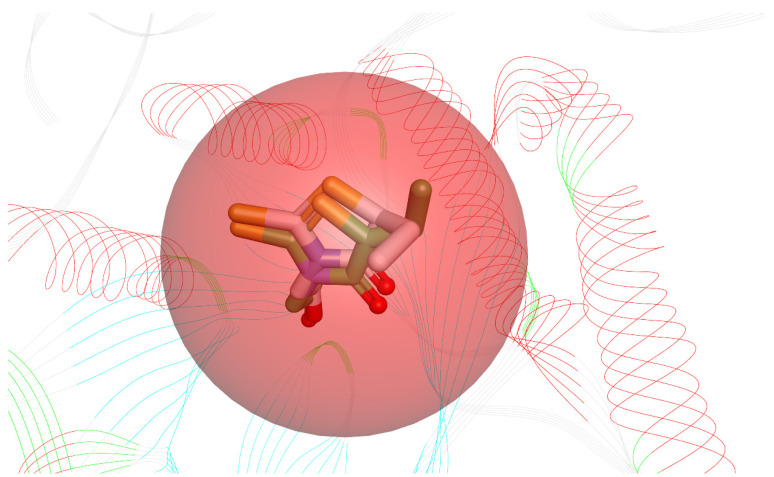
Superimposed bioactive structure of epalrestat bound in the binding pocket of ALR-2 (PDB ID: 4JIR) (green stick model) and the docked epalrestat (grey stick model) with an RMSD of 0.89 Å.

Before starting the docking procedure, the receptor protein was cleaned from co-crystal ligands and water molecules. The protein was prepared using “Prepare protein”protocol from DS to add hydrogen, repair missing atoms if any, and apply a CHARMm forcefield. The molecular docking simulations were run with the simulated annealing option set to True and the other options unchanged. To validate the docking software, we first docked the bound inhibitor-epalrestat into the binding pocket of ALR-2 (PDB ID: 4JIR) and calculated the RMSD between the ligand conformation of the docked and the x-ray crystal structure. The RMSD obtained was <1 Å, as shown in Figure 3 indicating the high accuracy of the CDOCKER software.

**Figure 4:**
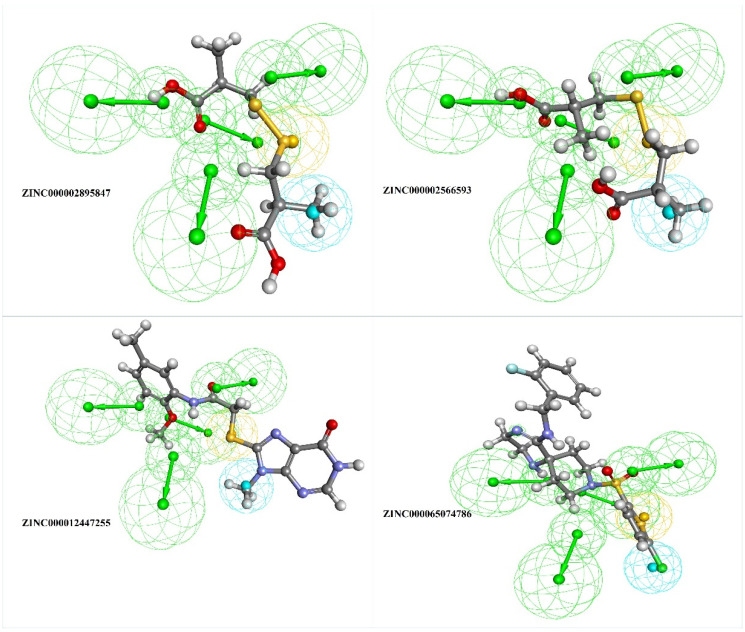
The top four filtered compounds aligned to the generated pharmacophore, where the green sphere indicates hydrogen bond acceptor, the blue sphere indicates hydrophobic, and the yellow sphere indicates sulfur interactions.

The top four selected natural compounds well aligned with the generated pharmacophore (Figure 4) were prepared for docking using “Prepare ligand” protocol from DS and were energy minimized.

On applying the CHARMm forcefield and selecting CFF as the ligand partial charge method, 10 random conformations for each ligand were selected. The best conformation having the highest negative CDOCKER score was further inspected for intra-molecular interactions. Table 6 describes the CDOCKER energy of the docked ligand and close intra-molecular interactions.

**Figure 5:**
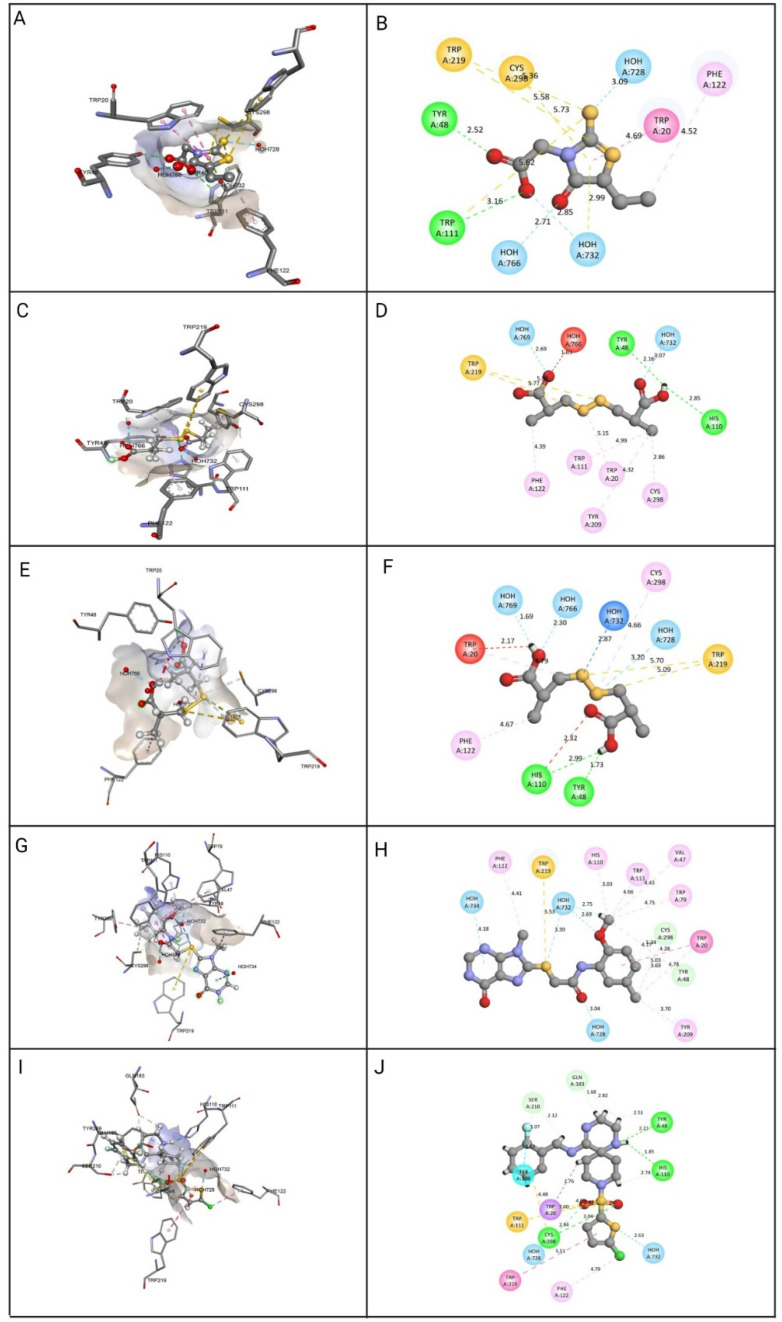
Molecular docking view of the compounds at the binding sites of ALR-2 (PDB ID: 4JIR). The stereo image of the docked complex containing the compound and receptor protein is presented on the left side, whereas the 2D view of the interactions between compounds and 4JIR is presented on the right side. Discovery Studio was used to construct the diagrams; hydrogen bonds are depicted in the green dashed line, hydrophobic interactions in the pink dashed line, and sulfur interactions in the yellow dashed line. The distance between them is displayed in angstroms. The amino acid residues in a protein structure were each given a three-letter code, and the compound is displayed in a ball-and-stick format. (**A**) epalrestat 3D view, (**B**) epalrestat 2D view, (**C**) ZINC000002895847 3D view, (**D**) ZINC000002895847 2D view, (**E**) ZINC000002566593 3D view, (**F**) ZINC000002566593 2D view, (**G**) ZINC000012447255 3D view, (**H**) ZINC000012447255 2D view, (**I**) ZINC000065074786 3D view, (**J**) ZINC000065074786 2D view.

The top four identified natural compounds were found to have higher -CDOCKER energy and -CDOCKER interaction energy than the epalrestat. Hydrogen bonding and hydrophobic interactions, which are weak intermolecular interactions, are important in maintaining energetically-favored ligands [33]. The compound ZINC000002895847 showed the highest -CDOCKER energy of 32.4309 and was found to form hydrogen bonds with the active site residue Tyr48, indicating the good binding affinity of the target-drug complex [34]. The compound also formed more hydrophobic interactions, contributing in ligand-receptor binding affinity and specificity [35]. The compound ZINC000002566593 secured the second highest -CDOCKER energy of 30.5462 and formed hydrogen and hydrophobic interactions with the residues in the binding pocket, including active site residues Tyr48, Trp219, and Phe122. The binding pose of compound ZINC000012447255 exhibiting -CDOCKER energy of 26.5493 was stabilized by hydrogen bonds and hydrophobic interactions, as shown in Figure 5. When it comes to establishing the conformation of the ligand that is most conducive to bioactivity, hydrophobic interactions play a crucial role. Because of these interactions, the selected site on the substrate is made more sterically accessible for drug metabolism [36]. The -CDOCKER energy of compound ZINC000065074786 was found to be close to epalrestat, however, it formed more hydrogen bonds and hydrophobic interactions with the binding site residues to aid with stability, affinity, and specificity.

## 4. Conclusions

The present study is an epalrestat-guided search for efficient ALR-2 inhibitors from a natural compound database using in silico pharmacophore-based screening, ADMET profiling, and molecular docking. These approaches were successfully used to determine the optimal binding mode of novel molecules necessary for ALR-2 inhibitory effects. Overall, four compounds were identified as possible ALR-2 inhibitors with drug-like features based on optimal binding modes, binding affinities, and critical interactions. In summary, the results demonstrate compounds derived from NuBBE_DB_ that preserve the pharmacophoric properties of epalrestat and are non-toxic with potential to inhibit the ALR-2 enzyme. In particular, ZINC000002895847, ZINC000002566593, ZINC000012447255, and ZINC000065074786 showed excellent binding affinity and specificity for ALR-2 that can be improved for the treatment of DPN. This study could pave the way for developing selective and safer ALR-2 inhibitors with a superior therapeutic profile than the current DPN treatments.

## Figures and Tables

**Table 1:** Virtually screened compounds from the natural compound database.

S. No.	Zinc ID	Pharmprint Frequency	Absolute Energy	Relative Energy
1.	ZINC000000839520	17,776	121.29	6.96097
2.	ZINC000002566593	16,450	168.778	16.3736
3.	ZINC000002895847	16,228	22.9699	13.843
4.	ZINC000004221776	12,899	22.1846	9.94709
5.	ZINC000004558041	10,411	123.539	14.3409
6.	ZINC000005189601	7480	53.2018	5.80273
7.	ZINC000005410978	7028	54.9265	2.95975
8.	ZINC000012447255	6756	75.4233	12.904
9.	ZINC000012483342	6741	44.9922	7.5057
10.	ZINC000013378346	5475	25.5627	14.7066
11.	ZINC000013380451	1254	40.2457	17.0317
12.	ZINC000018164733	1057	123.565	12.2268
13.	ZINC000038281168	1001	58.6725	8.66919
14.	ZINC000059585866	989	37.7033	1.78389
15.	ZINC000059586551	983	59.5097	6.99921
16.	ZINC000065074786	961	76.1116	1.88067
17.	ZINC000065259848	953	27.6677	7.31742
18.	ZINC000069482290	916	15.8298	5.27962
19.	ZINC000070455381	805	25.6468	8.67805
20.	ZINC000070686641	724	70.307	13.1053
21.	ZINC000070707266	720	33.4486	13.6762
22.	ZINC000084394823	703	62.7607	19.8907
23.	ZINC000085507556	661	14.4589	2.11996
24.	ZINC000085531653	660	27.514	8.40651
25.	ZINC000085593748	580	93.4406	18.6682
26.	ZINC000085809059	570	40.9	13.4514
27.	ZINC000097944195	562	130.569	9.30741
28.	ZINC000098022974	510	50.6495	12.9341
29.	ZINC000100393110	486	20.0607	9.37799
30.	ZINC000103571159	341	18.6231	13.8256
31.	ZINC000150349570	336	48.9624	5.99486
32.	ZINC000245238479	289	60.8105	5.31395
33.	ZINC000245296023	160	25.2688	10.6235
34.	ZINC000247764628	89	61.4826	17.7796

**Table 2:** LUDI scores for the virtually screened compounds.

S. No.	ZINC ID	Ludi_3
1.	ZINC000097944195	1328
2.	ZINC000070707266	1307
3.	ZINC000247764628	1280
4.	ZINC000012447255	1160
5.	ZINC000004558041	1013
6.	ZINC000100393110	989
7.	ZINC000065074786	964
8.	ZINC000004221776	879
9.	ZINC000000839520	875
10.	ZINC000070455381	832
11.	ZINC000085593748	801
12.	ZINC000245296023	799
13.	ZINC000245238479	783
14.	ZINC000098022974	747
15.	ZINC000253499410	719
16.	ZINC000012483342	705
17.	ZINC000065259848	655
18.	ZINC000103571159	590
19.	ZINC000248015717	523
20.	ZINC1533688 (Epalrestat)	490
21.	ZINC000085531653	456
22.	ZINC000070686641	433
23.	ZINC000059586551	427
24.	ZINC000038281168	404
25.	ZINC000059585866	403
26.	ZINC000013380451	367
27.	ZINC000084394823	360
28.	ZINC000069482290	349
29.	ZINC000013378346	324
30.	ZINC000085809059	299
31.	ZINC000002895847	289
32.	ZINC000002566593	282
33.	ZINC000005410978	264
34.	ZINC000005189601	264
35.	ZINC000018164733	237

**Table 3:** Drug-likeness properties of the compounds.

S. No.	ZINC ID	HBA	HBD	MW	ALogP	RoT	PSA
1.	ZINC000103571159	8	4	394.416	−1.043	1	132.28
2.	ZINC000084394823	6	5	180.156	−2.791	5	118.22
3.	ZINC000245238479	7	2	382.448	1.389	8	86.61
4.	ZINC000018164733	5	4	164.156	−1.903	4	97.99
5.	ZINC000100393110	7	2	446.56	2.897	9	113.71
6.	ZINC000000839520	8	3	376.43	1.797	6	132.99
7.	ZINC000012447255	8	2	359.403	1.586	5	122.91
8.	ZINC000013378346	0	0	238.457	2.483	7	101.2
9.	ZINC000002895847	4	2	238.324	1.77	7	125.2
10.	ZINC000065259848	5	2	328.45	1.476	3	117.08
11.	ZINC000248015717	7	3	374.449	0.996	4	146.43
12.	ZINC000253499410	6	2	507.706	3.533	5	144.46
13.	ZINC000004558041	9	2	381.404	1.747	7	144.1
14.	ZINC000004221776	9	6	375.421	−1.836	6	195.43
15.	ZINC000065074786	6	2	456.985	1.764	5	110.42
16.	ZINC000002566593	4	2	238.324	1.77	7	125.2
17.	ZINC000005410978	6	6	254.327	−5.934	8	177.23
18.	ZINC000012483342	4	3	253.317	0.693	2	90.01
19.	ZINC000098022974	11	0	382.367	−1.218	5	154.63
20.	ZINC1533688 (Epalrestat)	4	0	220.289	−0.279	3	112.04

Abbreviations: HBA: Hydrogen bond acceptor; HBD: Hydrogen bond donor; MW: Molecular Weight; PSA: Molecular polar surface area; RoT: Rotatable Bonds.

**Table 4:** In silico pharmacokinetic (ADMET) properties of the filtered compounds.

ZINC ID	Solubility	BBB Level	CYP 2D6	Hepatotoxic	Absorption	PPB	AlogP98	PSA 2D	BBB
ZINC000103571159	4	4	FALSE	TRUE	1	FALSE	−1.043	127.353	-
ZINC000084394823	5	4	FALSE	FALSE	3	FALSE	−2.791	121.378	-
ZINC000245238479	3	3	FALSE	FALSE	0	FALSE	1.388	86.281	−1.09
ZINC000018164733	5	4	FALSE	FALSE	1	FALSE	−1.903	100.562	-
ZINC000100393110	2	4	FALSE	FALSE	0	FALSE	2.897	105.998	-
ZINC000000839520	3	3	FALSE	FALSE	0	FALSE	1.991	109.004	−1.26
ZINC000012447255	3	3	FALSE	TRUE	0	FALSE	1.586	97.084	−1.2
ZINC000013378346	3	1	FALSE	TRUE	1	FALSE	2.483	0	0.61
ZINC000002895847	4	3	FALSE	FALSE	0	FALSE	1.77	76.232	−0.81
ZINC000065259848	3	3	FALSE	TRUE	0	TRUE	1.526	81.794	−0.97
ZINC000248015717	3	4	FALSE	FALSE	0	FALSE	0.996	123.278	-
ZINC000253499410	2	3	FALSE	FALSE	0	FALSE	3.009	88.515	−0.62
ZINC000004558041	3	4	FALSE	TRUE	0	FALSE	1.747	117.664	-
ZINC000004221776	4	4	FALSE	TRUE	3	FALSE	−1.786	162.994	-
ZINC000065074786	2	3	FALSE	TRUE	0	TRUE	2.229	74.897	−0.65
ZINC000002566593	4	3	FALSE	FALSE	0	FALSE	1.77	76.232	−0.81
ZINC000005410978	4	4	FALSE	FALSE	1	FALSE	−0.976	129.312	-
ZINC000012483342	3	3	FALSE	TRUE	0	FALSE	0.693	65.215	−0.97
ZINC000098022974	4	4	FALSE	TRUE	2	FALSE	−1.219	136.9	-
ZINC1533688	4	3	FALSE	TRUE	1	FALSE	−0.894	55.254	−1.30

Note: Aqueous solubility level (Solubility)-0: extremely low, 1: very low, 2: low, 3: good, 4: optimal; Blood-brain barrier (BBB) level-0: Very High, 1: High, 2: Medium, 3: Low, 4: Undefined; Intestinal absorption (Absorption)-0: Good absorption, 1: Moderate absorption, 2: Low absorption, 3: Very low absorption.

**Table 5:** Toxicity evaluation of the best-filtered compounds.

**ZINC ID**	ZINC000065259848	ZINC000065074786	ZINC000012447255	ZINC000245238479	ZINC000012483342	ZINC000253499410	ZINC000002895847	ZINC000002566593	Epalrestat
**Rat Female FDA**	Non-Carcinogen	Non-Carcinogen	Non-Carcinogen	Non-Carcinogen	Non-Carcinogen	Single-Carcinogen	Multi-Carcinogen	Multi-Carcinogen	Multi-Carcinogen
**Rat Male FDA**	Multi-Carcinogen	Non-Carcinogen	Non-Carcinogen	Non-Carcinogen	Non-Carcinogen	Non-Carcinogen	Non-Carcinogen	Non-Carcinogen	Multi-Carcinogen
**TD_50_ Mouse** **(mg/kg body weight/day)**	59.487	12.1147	101.749	13.831	53.7732	4.79071	107.426	107.426	82.6684
**TD_50_ Rat (mg/kg body weight/day)**	110.02	22.3253	11.4233	4.03207	5.3266	0.357734	95.4177	95.4177	30.0162
**Ames Prediction**	Non-Mutagen	Non-Mutagen	Non-Mutagen	Non-Mutagen	Non-Mutagen	Non-Mutagen	Non-Mutagen	Non-Mutagen	Non-Mutagen
**DTP Prediction**	Non-Toxic	Toxic	Non-Toxic	Toxic	Toxic	Toxic	Non-Toxic	Non-Toxic	Toxic
**Rat Oral LD_50_** **(g/kg body weight)**	0.55412	0.536551	5.11761	5.11263	0.19364	0.569967	1.06368	1.06368	2.71011
**Rat inhalation LC_50_ (mg/m3/h)**	317.172	739.959	6232.27	1761.33	519.752	245.134	2315.21	2315.21	5330.78
**Skin Irritancy**	Mild	None	None	None	None	Mild	None	None	Mild
**Ocular Irritancy**	Moderate	Moderate	Mild	None	Severe	Severe	Moderate	Moderate	Mild
**Aerobic Biodegradability**	Non-Degradable	Non-Degradable	Non-Degradable	-	Non-Degradable	Degradable	Degradable	Degradable	Degradable
**Fathead Minnow LC_50_** **(g/L)**	0.03	0.02	0.02	-	0.28	0	0.01	0.01	0.12
**Daphnia EC_50_ (mg/L)**	0.69	4.81	16.57	-	4.6	0.27	44.84	44.84	21.28

**Table 6:** Close intra-molecular interaction of the top pharmacophores and ALR-2.

Compound	Intra-Molecular Interactions	-CDOCKER Energy	-CDOCKER Interaction Energy
Epalrestat	Hydrogen Bond: HOH728, HOH732, HOH766, Tyr48, Trp111. Other: Cys298, Trp111, Trp219. Hydrophobic: HOH732, Trp20, Phe122.	13.4173	25.039
ZINC000002895847	Hydrogen Bond: HOH728, HOH769, Tyr48, His110. Other: Trp219. Hydrophobic: Cys298, Trp20, Trp111, Phe122, Phe209.	32.4309	29.8047
ZINC000002566593	Hydrogen Bond: HOH732, HOH728, HOH766, HOH769, Tyr48, His110. Other: Trp219. Hydrophobic: Cys298, Trp20, Phe122.	30.5462	26.683
ZINC000012447255	Hydrogen Bond: HOH732, HOH728, HOH734, HOH732, Tyr48, Cys298. Other: Trp219. Hydrophobic: Trp20, Tyr48, Val47, Cys298, Trp79, His110, Trp111, Phe122, Tyr209.	26.5493	47.9781
ZINC000065074786	Hydrogen Bond: HOH732, HOH728, Tyr48, Cys298, his110, Gln183, Tyr48, Ser210. Other: Trp20, Cys298, Trp111. Hydrophobic: Trp20, Trp219, Phe122.	13.6588	32.9367

## Data Availability

Not applicable.
